# Detection of Image Seam Carving Using a Novel Pattern

**DOI:** 10.1155/2019/9492358

**Published:** 2019-11-11

**Authors:** Ming Lu, Shaozhang Niu

**Affiliations:** ^1^Beijing Key Lab of Intelligent Telecommunication Software and Multimedia, Beijing University of Posts and Telecommunications, Beijing 100876, China; ^2^School of Computer Science and Software Engineering, University of Science and Technology Liaoning, Anshan 114051, China

## Abstract

Seam carving is an excellent content-aware image resizing technology widely used, and it is also a means of image tampering. Once an image is seam carved, the distribution of magnitude levels for the pixel intensity differences in the local neighborhood will be changed, which can be considered as a clue for detection of seam carving for forensic purposes. In order to accurately describe the distribution of magnitude levels for the pixel intensity differences in the local neighborhood, local neighborhood magnitude occurrence pattern (LNMOP) is proposed in this paper. The LNMOP pattern describes the distribution of intensity difference by counting up the number of magnitude level occurrences in the local neighborhood. Based on this, a forensic approach for image seam carving is proposed in this paper. Firstly, the histogram features of LNMOP and HOG (histogram of oriented gradient) are extracted from the images for seam carving forgery detection. Then, the final features for the classifier are selected from the extracted LNMOP features. The LNMOP feature selection method based on HOG feature hierarchical matching is proposed, which determines the LNMOP features to be selected by the HOG feature level. Finally, support vector machine (SVM) is utilized as a classifier to train and test by the above selected features to distinguish tampered images from normal images. In order to create training sets and test sets, images are extracted from the UCID image database. The experimental results of a large number of test images show that the proposed approach can achieve an overall better performance than the state-of-the-art approaches.

## 1. Introduction

### 1.1. Motivation

**Figure 1:**
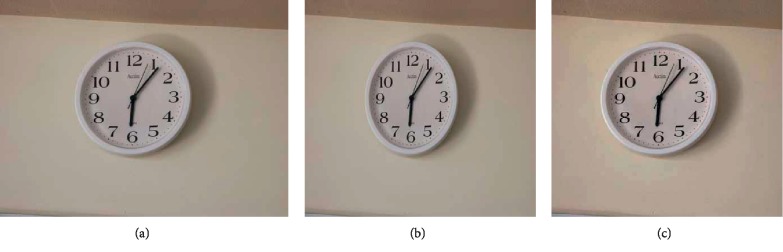
Comparison of seam carving and conventional resizing. (a) The original image from UCID (ucid00542.tif). (b) The image with its width reduced by 20% by conventional resizing. (c) The image with its width reduced by 20% by seam carving.

Among many applications of digital image processing, image resizing is a very common and important one. Conventional technology of image resizing often causes image content to be deformed while changing the aspect ratio of the image and then affects image display effect. In 2007, Avidan and Shamir proposed a content-aware image resizing (CAIR) [1], among which seam carving technology is most accepted widely. The technology avoids distortion of image details while resizing digital images, by removing the optimal seams with low energies and preserving regions with high energies in the image. As shown in Figure 1, after the width of the image is reduced by 20%, the wall clock in Figure 1(b) has undergone obvious deformation, but the one in Figure 1(c) keeps its original size. Because of the particular advantages, seam carving technology develops rapidly and is widely used. However, seam carving technology can also be deliberately used to change semantic content in the image. It is convenient to tamper with the content of the image deliberately or even maliciously. Therefore, it is an urgent requirement to design a more efficient method to detect seam carving operation for image forensics.

### 1.2. Related Work

At present, there are some approaches for detection of seam carving. Lu and Wu [2] proposed an active forensics method, Hash-based forecasting and discrimination method, which has some limitations in application. Forensic hash can be easily deleted, and that must be built in advance because of the active forensic techniques. Sarkar et al. [3] proposed a detection method based on the Markov feature; the method only considers the correlation between adjacent pixels, but ignores the correlation between nonadjacent pixels. So, the accuracy of detection is not high. In this regard, Sheng et al. [4] proposed a detection method based on extended Markov characteristics. The Markov features which can describe the correlation between nonadjacent pixels with different step sizes were added, and the detection effect was improved obviously. In recent years, they also proposed a detection method based on Benford's law [5]. Fillion and Sharma [6] proposed a detection method which only has good recognition performance for the clipped images with large resizing ratio; multifeature fusion method was used to detect tampering. Wei et al. [7] proposed a patch analysis approach to detect images seam carving. The approach constructs Markov features by connecting patches in subdirection, vertical direction, and diagonal direction. Ryu et al. [8] proposed a detection approach based on the noise and energy bias features to detect traces of seam carving and seam insertion and obtain a better detection result. In addition, the detection approach of seam carving uses a local descriptor describing image texture [9, 10], which has a better detection effect than the Markov feature. Ye et al. [11] introduced LDP (local derivative pattern) to detect tampering, and the effect is improved compared with previous methods. The content-adaptive detection method proposed in reference [12] can locate tampered areas accurately. Zhang et al. [13] proposed a detection approach based on ULBP (uniform local binary patterns) for images seam carving; compared with Wei et al. [7] and Ryu et al. [8], it improves the performance greatly. The detection of seam carving can be seen as a scenario application in the whole field of digital image forensics. Some more recent works about image forgery detection also opens up forensic ideas for the detection of seam carving, such as a robust copy-move forgery detection algorithm proposed by Chen et al. [14], which considers the FrQZMs as a feature, and a modified PatchMatch algorithm as a feature-matching algorithm. After that, in order to generalize the conventional fractional cosine transforms (FrCT) to quaternion signal processing in a holistic manner, Chen and his team proposed fractional quaternion cosine transforms (FrQCT) and then proposed a new color image copy-move forgery detection algorithm based on FrQCT and modified PatchMatch matching algorithm, which is used to evaluate the performance of the proposed FrQCT [15].

### 1.3. Main Contribution

Although the existing approaches have certain efficiency, the accuracy and performance indicators are still not ideal and lack robustness, and there is still a large room for improvement. If the important properties and inherent features which are different from other image tampering are fully exploited in the detection of seam carving, the detection accuracy can be further improved. The distribution of the magnitude levels for the pixel intensity differences in the local neighborhood is changed when an image is subjected to seam carving. Therefore, it is the key issue to detect image seam carving efficiently on how to accurately describe the change of the magnitude levels for local neighborhood pixels. In this regard, we propose an effective and novel image pattern descriptor, we term local neighborhood magnitude occurrence pattern (LNMOP), which can be used to describe the distribution of pixel intensity differences by counting up the number of magnitude levels. However, it is dissimilar from the traditional descriptors such as local binary pattern (LBP) [16]. LBP is only a sign pattern, not a magnitude pattern, lacking of the magnitude information among neighboring pixels, and is sensitive to noise and has poor robustness. The antinoise ability and magnitude recognition ability are significantly enhanced in LNMOP.

In this paper, an image tampering detection approach using LNMOP features for seam carving is proposed, which not only overcomes the insensitivity of traditional Markov feature in detecting small-scale tampering but also avoids the problem that the LBP feature confuses texture region and smooth region due to noise and then improves the performance as a whole. Firstly, the HOG feature level is determined, and then the final features are selected according to the HOG feature level from the extracted LNMOP features. Finally, the classifier is trained and tested by the final features.

The main contributions of the paper as follows:

1. A new local pattern called local neighborhood magnitude occurrence pattern (LNMOP) has been proposed to extract the local features from the distribution of magnitude levels for the pixel intensity differences in the local neighborhood that fulfil the lack of magnitude information required for a local descriptor.
2. An approach for the detection of seam carving based on LNMOP and HOG has been proposed, which uses different detection radii according to the HOG features. The performance of detection is also better for relatively smooth region.
3. In view of the selection of LNMOP features, an LNMOP feature selection algorithm based on HOG feature hierarchical matching is proposed, which reduces the dimension of LNMOP and reduces the computation of classifier effectively.

The paper is organized in the following manner: In Section 1, introduction of the problem, including motivation, related work, and the main contribution of the paper, have been described.  Section 2 briefly overviews seam carving process. The proposed image feature is explained in Section 3. The proposed system framework for the detection is explained in Section 4.  Section 5 demonstrates the experimental results and discussions. Finally, the work is concluded in Section 6.

## 2. Overview of Seam Carving

Seam carving is a content-aware image resizing technology proposed by Avidan in 2007 [1]. It adjusts the aspect ratio of images by removing a certain number of seams with least importance in the image, while preserving the important region of interest from distortion in the image. A seam is an eight-connected path of low-energy pixels passing through the image from top to bottom or from left to right. An energy function is defined as in equation (1); the larger the function value is, the less likely the region belongs to the smooth region, and the more important the content of the image is.

(1) $$\begin{array}{c} e\left(I\right)=\left|\frac{\partial }{\partial x}I\right|+\left|\frac{\partial }{\partial y}I\right|, \end{array}$$

where *I* represents an image, i.e., *I*(*x*, *y*); *x* and *y* are the row coordinate and column coordinate, respectively; and *e* is the energy function.

A series of pixels with the lowest energy value calculated by equation (1) can form a connection path vertically or horizontally, which is called a “seam.” For an *m* × *n* image, the vertical seam is defined as follows:

(2) $$\begin{array}{c} \begin{array}{cc} & S^{x}={\left\{S_{i}^{x}\right\}}_{i=1}^{m}={\left\{i,\;x\left(i\right)\right\}}_{i=1}^{m},\\ \text{s.t.} & \forall i,\left|x\left(i\right)-x\left(i-1\right)\right|\leq 1, \end{array} \end{array}$$

where *S*_*i*_^*x*^ is the location of each pixel in the seam and *x*(*i*) is the column coordinate corresponding to the pixel in row *i*. Similarly, the horizontal seam is defined as follows:

(3) $$\begin{array}{c} \begin{array}{cc} & S^{y}={\left\{S_{j}^{y}\right\}}_{j=1}^{n}={\left\{j,\;y\left(j\right)\right\}}_{j=1}^{n},\\ \text{s.t.} & \forall j,\left|y\left(j\right)-y\left(j-1\right)\right|\leq 1, \end{array} \end{array}$$

where *S*_*j*_^*y*^ is the location of each pixel in the seam and *y*(*i*) is the row coordinate corresponding to the pixel in column *j*. Pixels in the vertical seam can be expressed as follows:

(4) $$\begin{array}{c} I_{s}={\left\{I\left(S_{i}\right)\right\}}_{i=1}^{m}={\left\{I\left(i,x\left(i\right)\right)\right\}}_{i=1}^{m}. \end{array}$$

Similarly, pixels in the horizontal seam can be expressed as follows:

(5) $$\begin{array}{c} I_{s}={\left\{I\left(S_{j}\right)\right\}}_{j=1}^{n}={\left\{I\left(j,x\left(j\right)\right)\right\}}_{j=1}^{n}. \end{array}$$

Seam carving accomplishes image resizing by selecting seam and removing pixels at seam. If the image is reduced horizontally, we must consider how to select the vertical seam. In order to achieve the best display effect, we must choose the optimal seam. Such seam contains the lowest energy of the pixel set. According to equation (4), the optimal seam can be defined as

(6) $$\begin{array}{c} S^{*}=\underset{s}{\min}\left\{E\left(s\right)\right\}=\underset{s}{\min}\left\{\overset{m}{\underset{i=1}{\sum }}e\left(I\left(S_{i}\right)\right)\right\}, \end{array}$$

where *E*(*s*) represents the cumulative energy and *S*^*∗*^ is the optimal seam. Similarly, if the image is reduced vertically, the optimal seam is defined as

(7) $$\begin{array}{c} S^{*}=\underset{s}{\min}\left\{E\left(s\right)\right\}=\underset{s}{\min}\left\{\overset{m}{\underset{j=1}{\sum }}e\left(I\left(S_{j}\right)\right)\right\}. \end{array}$$

The selection of optimal seam can be achieved by dynamic programming algorithm. In this paper, vertical seam is taken as an example. For any pixel point (*i*, *j*) on seam, from the second row to the last row, there are only three pixels (*i* − 1, *j* − 1), (*i* − 1, *j*), and (*i* − 1, *j* + 1) adjacent to the previous line. The cumulative minimum energy *M*(*i*, *j*) should satisfy

(8) $$\begin{array}{c} M\left(i,j\right)=e\left(i,j\right)+\min\left(M\left(i-1,j-1\right),M\left(i-1,j\right),M\left(i-1,j+1\right)\right). \end{array}$$

**Figure 2:**
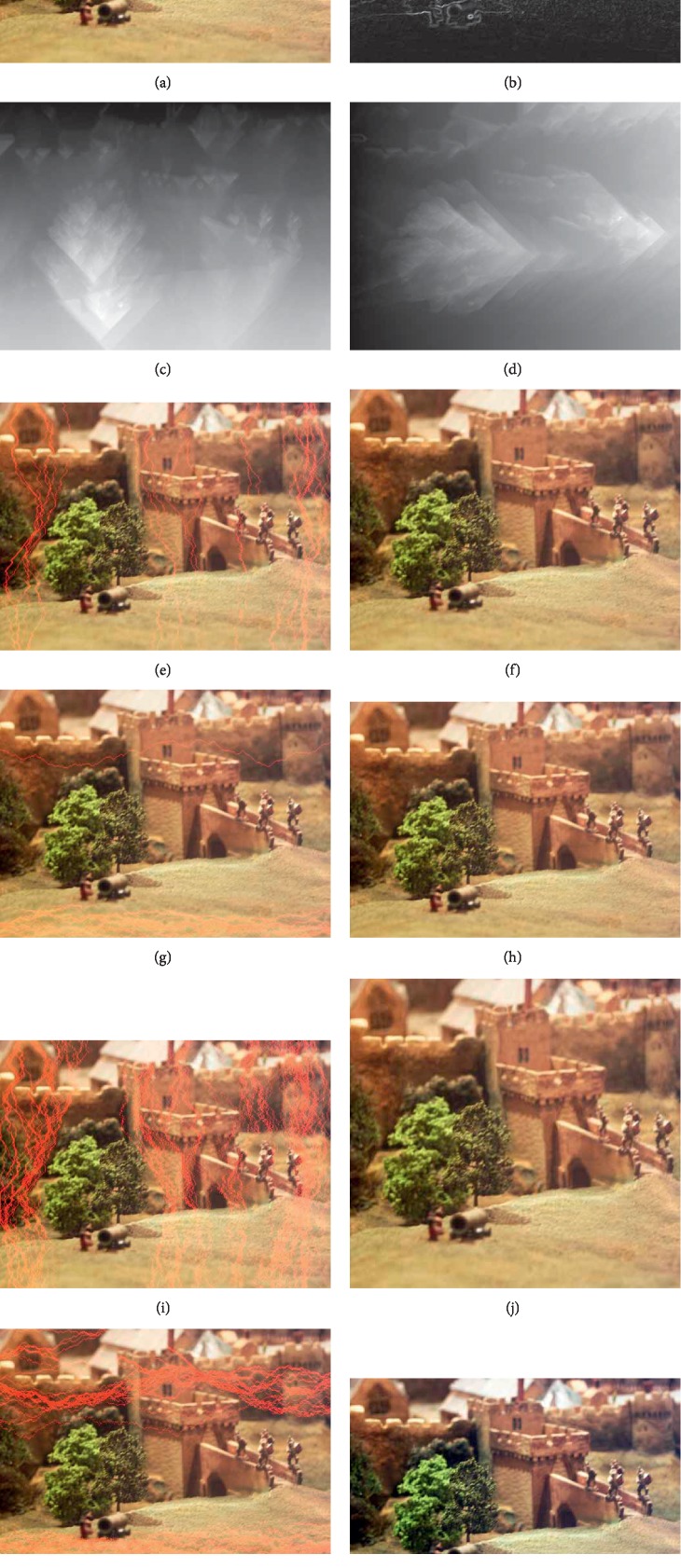
Demonstration of seam carving. (a) The original image from UCID (ucid00066.tif); (b) L1-norm gradient map; (c) cumulative energy map in the vertical direction; (d) cumulative energy map in the horizontal direction; (e) the original image with 5% vertical seams in red; (f) seam-carved image with 5% reduced width; (g) the original image with 5% horizontal seams in red; (h) seam-carved image with 5% reduced height; (i) the original image with 20% vertical seams in red; (j) seam-carved image with 20% reduced width; (k) the original image with 20% horizontal seams in red; (l) seam-carved image with 20% reduced height.

When the last row is searched, the position where there is the minimum energy of the last row is taken as the ending location of the optimal vertical seam. The optimal seam is found by back-tracking from the last row upward, and then it is removed. By performing the above process recursively, the width of the image can be reduced. Similarly, the height of the image also can be reduced in the same way. As shown in Figure 2, the seam optimally appears in dark areas of the gradient map and cumulative energy map in the corresponding direction. So, the seam selection follows the principle of low energy priority. It also shows that there is no visual distortion in the salient areas of images with different aspect ratios. It is difficult for the human eye to distinguish whether an image has been tamped or not.

In summary, seam carving is an excellent solution for image resizing because it has content awareness. However, due to the missing of pixels, image content will inevitably change; although the visual impact is very limited or even not obvious, the relationship between neighborhood pixels will also change. In particular, it is more evident that the change of the magnitude distribution of pixel intensity differs in the local neighborhood. However, as mentioned before, the seam optimally appears in the region with smaller gradients, which is the relatively smooth region. The texture features of relatively smooth regions are very weak, and the magnitude features of intensity difference are also very weak. It is difficult to detect the change of the magnitude of intensity differences in smoothed regions. Compared with the detection of seam carving with the large resizing ratios, it is more difficult to detect seam carving with the small resizing ratios, due to the fact that most seams pass through the relatively smooth region for seam carving with the small resizing ratios. Therefore, we present a novel feature pattern which depicts variation of intensity differences among pixels in different radius neighborhoods so as to reveal the trace of seam carving in the relatively smooth region.

## 3. The Proposed Image Feature

### 3.1. Local Neighborhood Magnitude Occurrence Pattern

The magnitude information is also one of the important features of local neighborhood pixel correlation. Most of the existing descriptors are not strong enough to describe the magnitude of the difference between the pixel and neighborhood pixels in the relatively smooth region. To address this problem, we considered the local occurrences of magnitude information in a local neighborhood region. In the present work, a new feature extraction method called local neighborhood magnitude occurrence pattern (LNMOP) is proposed. Just as its name implies, the method extracts local features based on the difference magnitude occurrence between the central pixel and neighborhood pixels and forms a binary pattern to represent each pixel in the image. A local magnitude occurrence binary pattern is generated for each pixel of the image, following the three steps:

First, the magnitude levels are determined according to the threshold *λ*. Second, the number of occurrences for each magnitude level in local neighborhoods is represented in binary form. Finally, the binary form for each magnitude level is concatenated to generate a single pattern for that pixel. By constructing such descriptors, a binary pattern can be generated for each pixel.

Let *I* be an image of size *m* × *n*, and *P*(*x*, *y*) represents any pixel in the neighborhood with radius *r*, and the number of occurrences for the magnitude level *l* in the local neighborhood of pixel *P*(*x*, *y*) is represented as **ℤ**_(*x*, *y*)_^*i*,*r*^, where *x* ∈ [*r*+1, *m* − *r*] and *y* ∈ [*r*+1, *n* − *r*]. The magnitude level *l* is given as *l* ∈ [1, *λ*], *λ*=2*r*+1. The threshold is determined by dynamic adjustment in practical application, and it should not be too large or too small. If it is too small, it will affect the ability of magnitude description. On the contrary, this will increase the dimension of features and increase the complexity. With the increase of local neighborhood radius *r*, the difference of intensity between a pixel and its neighborhood may also increase, which requires more magnitude levels *l*, so the threshold *λ* has a certain relationship with radius *r*, and the maximum possible value of *r* is ⌊[min(*m*, *n*)/2]⌋.

The binary pattern of length *k* for the magnitude level *l* in the local neighborhood with the radius *r* of pixel *P* (*x*, *y*) is defined as

(9) $$\begin{array}{c} \Psi_{\left(x,y\right)}^{l,r}=\varsigma \left({\mathbb{Z}}_{\left(x,y\right)}^{l,r},k\right), \end{array}$$

where *ς*(*a*, *b*) uses the binary numbers of length *b* to represent decimal value *a*, *ℤ*_(*x*, *y*)_^*l*,*r*^ ∈ [0, (2*r*+1)^2^]. The length *k* of binary pattern for each magnitude level should be large enough to avoid the problem of insufficient length in the decimal-to-binary conversion. The value of *k* is determined by the maximum possible value of *ℤ*_(*x*, *y*)_^*l*,*r*^, which is (2*r*+1)^2^. As a result, *k* is given as *k*=⌈log_2_(2*r*+1)⌉.

The local neighborhood magnitude occurrence pattern for pixel *P*(*x*, *y*) in its radius *r* neighborhood is generated by concatenating the patterns for each magnitude level *l* and defined as

(10) $$\begin{array}{c} {\text{LNMOP}}_{\left(x,y\right)}^{r}=\left\{\Psi_{\left(x,y\right)}^{1,r},\Psi_{\left(x,y\right)}^{2,r},\ldots ,\Psi_{\left(x,y\right)}^{\lambda ,r}\right\}. \end{array}$$

**Figure 3:**
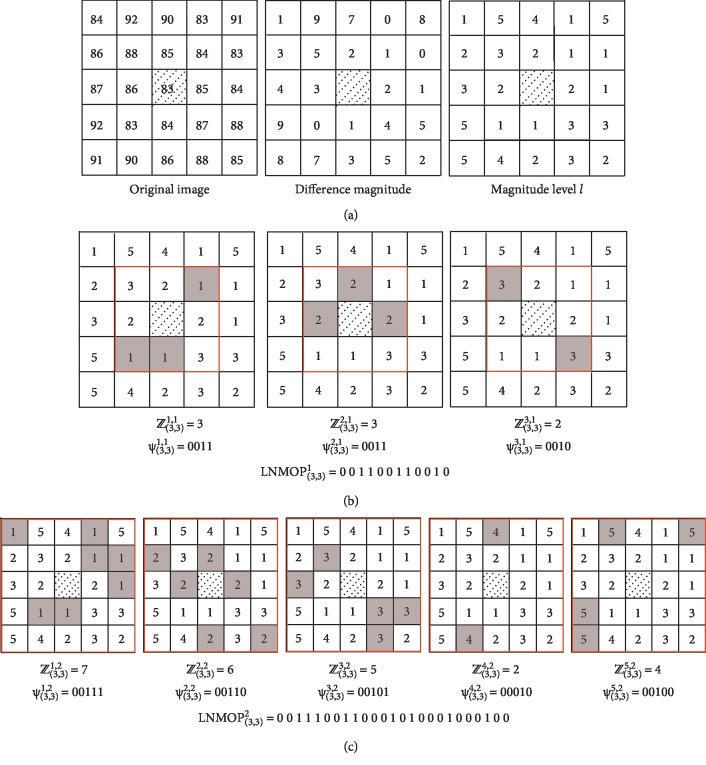
The local neighborhood magnitude occurrence pattern examples. (a) Illustration to compute the magnitude level *l* from the original image. (b) The LNMOP pattern for *P* (3, 3) in its radius *r* = 1 neighborhood. (c) The LNMOP pattern for *P* (3, 3) in its radius *r* = 2 neighborhood.

In Figure 3, an example of LNMOP calculation has been demonstrated; the LNMOP pattern for the center pixel *P* (3, 3) of the neighborhood with *r* values of 1 and 2 has been obtained, and the calculation process of that is very clear as shown in Figure 3.  Figure 3(a) shows the whole process of calculation of the magnitude level *l* from the original image; as an example, the intensities of the center pixel *P* (3, 3) and its neighborhood pixels is shown in the first graph, and the second graph illustrates the magnitudes of the intensity difference between the center pixel *P* (3, 3) and each neighborhood pixel. Then, the interval of magnitude distribution is determined, and the interval is divided into levels, and the number of magnitudes belonging to a certain level is counted up; thus, we get the result shown in the third graph. As shown in Figure 3(b), the value of *r* is considered as 1 so that the maximum possible value of *ℤ*_(3,3)_^*l*,1^ becomes 9, the value of length *k* will be 4, and the numbers of occurrences of the magnitude level *l* are 3, 3, and 2 for *l* = 1, 2, and 3, respectively. Similarly, it is also computed in Figure 3(c) for the same example of Figure 3(b); the value of length *k* will be 5 so that the maximum number of occurrence can be represented in the *k*-bit binary form, and the numbers of occurrences of the magnitude level *l* are 7, 6, 5, 2, and 4 for *l* = 1, 2, 3, 4, and 5, respectively.

### 3.2. LNMOP Descriptor Normalization

A local neighborhood magnitude occurrence pattern is generated for each pixel of the image in its local neighborhood with radius *r*. Then, the length of the binary pattern for each pixel is *k* × *λ*, and there are *m* × *n* pixels in the image; if each binary pattern is simply concatenated into a single pattern, then the size of the final pattern will be *m* × *n* × *k* × λ, which is too high for efficient storage and operation. To address this problem, we adopted bitwise addition of each binary pattern, and the process of which is given by the descriptor tol (*s*) as follows:

(11) $$\begin{array}{c} \text{tol}\left(s\right)=\overset{m-r}{\underset{i=r+1}{\sum }}\overset{n-r}{\underset{j=r+1}{\sum }}{\left.{\text{LNMOP}}_{\left(i,j\right)}^{r}\right|}_{s},\;\forall s=\left[1,k\times \lambda \right]. \end{array}$$

The dimensions of “tol (*s*)” depend on the size of the image. For high resolution images, the values of tol (*s*) elements will be larger, i.e.,

(12) $$\begin{array}{c} \text{tol}\left(s\right)\propto \left(m\times n\right). \end{array}$$

In order to avoid too large dimensions of the descriptor in practical application, we normalize the descriptor to make it independent of image size. The normalized local neighborhood magnitude occurrence pattern descriptor is represented as

(13) $$\begin{array}{c} {\text{LNMOP}}_{\text{norm}\left(s\right)}=\frac{\text{tol}\left(s\right)}{\sum_{t=1}^{k\times \lambda }\text{tol}\left(t\right)},\;\forall s=\left[1,k\times \lambda \right]. \end{array}$$

Algorithm 1 gives a pseudocode description for the construction process of LNMOP.

### 3.3. The Contribution of LNMOP in Detection

**Figure 4:**
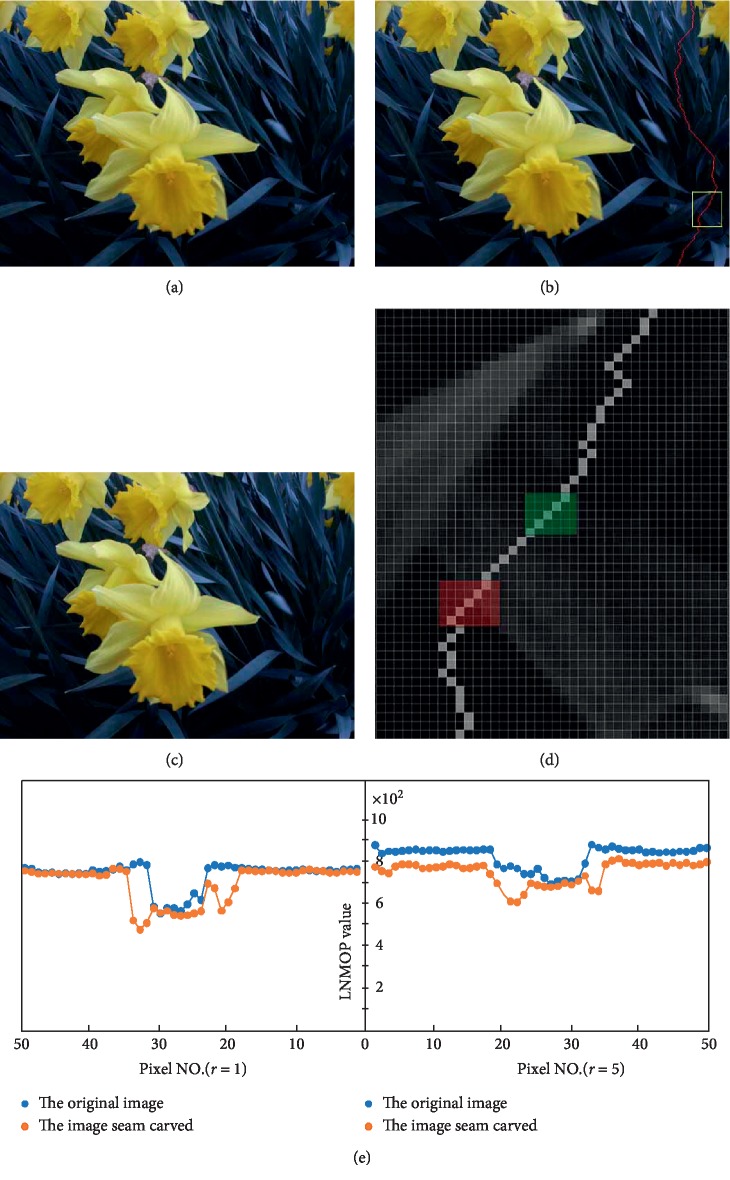
The effect of seam carving towards the LNMOP values. (a) The original image from UCID (ucid00081.tif); (b) the original image with a vertical seam; (c) the image with a removed seam; (d) the region with a yellow rectangular box in Figure 4(b); (e) the difference of the LNMOP values for the pixels along the seam between the original image and the image seam carved, in the region shown in Figure 4(d).

Let us review the process of seam carving. When a vertical seam is deleted, the right pixel adjacent to the seam in each row moves one pixel to the left to fill the deleted seam path. Similarly, the horizontal seam is deleted, and the adjacent pixel beneath the seam in each column moves upwards one pixel to fill the deleted seam path. Whether a vertical or horizontal seam is removed, it will affect the magnitude of the pixel intensity difference. Therefore, the distribution of the magnitude levels in the local neighboring along this seam path will be obviously changed. So, compared with the LNMOP values of the original image, the LNMOP values of the image seam carved will be modified. It can be seen that LNMOP can be used as an effective feature for the detection of seam carving.  Figure 4 presents the contribution of LNMOP for the local neighborhood pixels in detecting seam carving.

In order to contrast and analysis, we select a small local region along the seam (the region marked with a yellow box in Figure 4(b)). Then, the small region is enlarged (as shown in Figure 4(d)) to facilitate the study of changes in the LNMOP values of pixels along the seam caused by the deletion of the seam. As can be seen from Figure 4(d), the seam passes through two special regions (the regions covered by red and green masks in the figure), which contain the edges of the object. We know that the gradient is large, and the intensities of pixels change fast in the edge region. Therefore, in the edge region, the magnitude of the pixel intensity difference also changes considerably after the seam is removed; furthermore, the change of LNMOP value caused by the deletion of the seam is also more obvious, and the expected effect can be achieved by using small radius detection in the detection of seam carving. Compared with edge regions, the change of LNMOP values along the seam in smooth regions is not significant, so large radius detection is needed.  Figure 4(e) confirms the above discussion. In the detection of seam carving, the large radius detection will increase computational complexity, and the specific detection radius *r* is determined by the gradient feature of the image, which is assisted by the histogram of oriented gradient (HOG) [17] feature (see Section 4 for details).

## 4. The Proposed Framework for the Detection of Seam Carving

**Figure 5:**
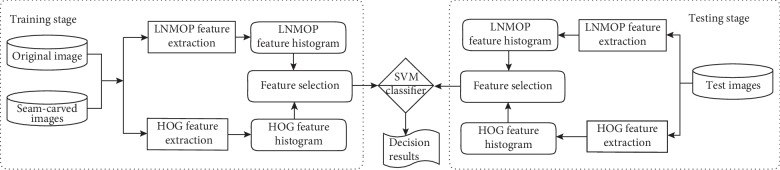
The proposed detection framework of image seam carving forensics.

In order to detect efficiently whether an image is subjected to seam carving or not, a forensic approach is proposed in this paper. The whole framework of the approach is shown in Figure 5. The framework consists of two parts, one is the training of classifiers and the other is the detection of digital images. In the training stage, a digital image is converted into a gray image, and then the statistical features of LNMOP and HOG are extracted by blocks and expressed by histogram. Then, the LNMOP features were screened according to the HOG level to generate the final features, and it makes the detection more accurate and efficient. Finally, the final features are used to train and test classifiers. Whether an image is seam carved or not is a binary classification problem. Therefore, the support vector machine (SVM) [18] is used as a classifier here. When the number of training samples is constant, the SVM classifier can provide better classification performance.

### 4.1. Feature Selection

As mentioned above, large radius detection is very effective for smoothing regions, but it has high computational complexity. For edge regions, only small radius detection is needed, which can improve detection efficiency; HOG features [17] can help us balance. We can use HOG feature to screen LNMOP features for classifiers. In order to further improve the detection accuracy and efficiency, this paper proposes an LNMOP feature selection algorithm based on HOG feature hierarchical matching.

The main idea is that we divide the original image into blocks of equal size and nonoverlapping each other, extract HOG features from each block, and classify HOG features for each block according to the size of detection radius. Then, according to the different detection radii, LNMOP features are extracted from each block for several times, and multilevel LNMOP features are obtained. Finally, the LNMOP feature matching the HOG feature level in each block is selected and concatenated into the final features for the classifier.

The pseudocode of the algorithm is described as follows.

**Figure 6:**
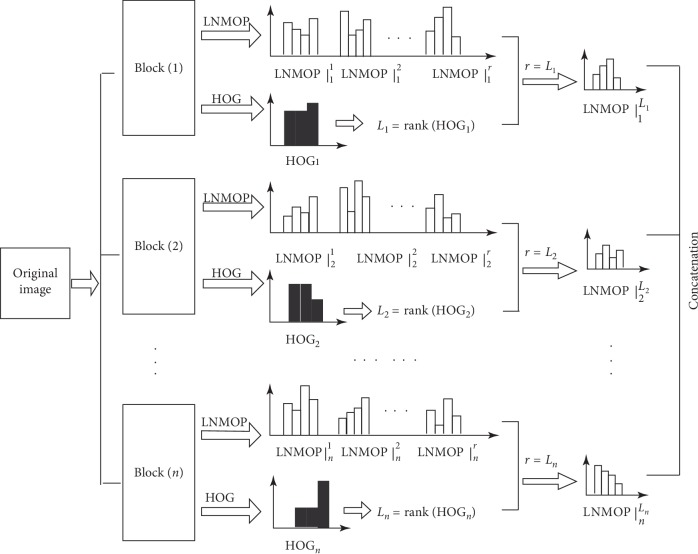
The flowchart of Algorithm 2.

The flowchart of Algorithm 2 is shown in Figure 6.

### 4.2. Classifier Training

In this paper, LIBSVM [19] is used as a classifier, radial basis function (RBF) is used as the kernel function. The RBF kernel function can solve the linear inseparable problem, and the number of parameters is less than polynomial kernel function, so it is easy to adjust parameters. The optimal parameters *c* and gamma are obtained by grid search algorithm. In order to avoid overfitting and improve generalization ability of classifiers, the generalization error is reduced, and the accuracy of classifiers is improved by minimizing the empirical risk and confidence range.

## 5. Experimental Results and Analysis

In order to verify the performance of the proposed seam carving detection method, a series of the experimental results are reported. The experimental samples are selected from UCID (Uncompressed Color Image Database, the second edition) [20] as the benchmark. It contains 1338 uncompressed real color TIFF images with rich contents, such as human, scenery, building and animals, and so on, and the size of the images is 512 × 384 or 384 × 512. The experiments are conducted with a personal computer, whose configuration parameters are Intel (R) Core (TM) I3 CPU 2.4 GHz, Memory 8 GB, and Windows 7 (64 bits). The detector is implemented with the software platform Matlab2015a.

In the experiment, 1000 images were randomly selected as the training set of original images, and the remaining 338 images were used as the test set of original images, to verify the effect of seam carving detection. Considering the influence of different resizing ratios on the detection results, the experiments are conducted in the following three groups: (1) the detection of images with small resizing ratios; (2) the detection of images with large resizing ratios; (3) the detection of images with mixed resizing ratios. Therefore, it is necessary to prepare three groups of training sets and test sets for tampered images. However, there is no publicly available seam-carved image database, which can be directly utilized for detection of seam carving. Thus, we create tampered image datasets by ourselves. In the first group experiment, there are 1000 original images in the training set, and each image in the training set is reduced horizontally by seam carving method [1]. The resizing ratios range from 3% to 11% with a step size of 2%; 5000 seam-carved images are generated as the training set of tampered images. 1690 seam-carved images as the test set of tampered images are generated from 338 original images in test set by the same method. In the second group experiment, the resizing ratios range from 10% to 50% with a step size of 10%. 5000 seam-carved images as the training set of tampered images and 1690 seam-carved images as the test set of tampered images are generated using the same method as before. In the third group experiment, the training set of tampered images and the test set of tampered images are composed of the corresponding image sets in the first and second group experiments, respectively. In order to verify the performance of the proposed approach, considering the hardware and software environment used in the experiment, the approaches in reference [13] and reference [8] are selected to compare with the one in this paper.

### 5.1. The Detection of Images with Small Resizing Ratios

Table 1 shows the performance of the detection for images seam carving with small resizing ratios. True positive rate (TPR) indicates the proportion of successfully detected tampered image samples to the whole samples to be detected. True negative rate (TNR) indicates the proportion of successfully detected normal image samples to the whole samples to be detected. Accuracy is the proportion of successfully detected samples (including positive and negative ones) to the whole samples to be detected.

As can be seen from Table 1, with the increase of the resizing ratio, the true positive rate, the true negative rate, and the accuracy rate are significantly improved, which reflects that the resizing ratio of image has a great impact on the performance of detection. Therefore, it is not meaningful to compare with the results of the detection for different resizing ratios, which is also the reason for different resizing ratios were tested separately in this experiment. The true positive rate is mostly lower than the true negative rate, which shows that the ability to detect tampered images correctly is weaker than the ability to detect normal images correctly.

As shown in Table 2, compared with Ryu et al. [8], Zhang et al. [13] can describe the small local texture change of the seam-carved image. Therefore, the detection accuracy of Zhang et al. [13] is higher than Ryu et al. [8] on the same resizing ratio. However, the proposed approach can achieve the highest detection accuracy among the three detectors.

### 5.2. The Detection of Images with Large Resizing Ratios

Table 3 shows the detection performance for the images seam carving with large resizing ratios; it is apparent that the true positive rate is higher than the true negative rate, which shows that the ability to detect tampered images correctly is better than the ability to detect normal images correctly. Compared with the detection for images with small resizing ratios, it is easier to detect seams correctly. The detection accuracy for images with large resizing ratios is very high. When the resizing ratio is more than or equal to 10%, the accuracy reaches more than 90.12%. This is because when an image is resized with large resizing ratio by seam carving, the magnitude level of the pixel intensity difference in the local neighborhood can change significantly.

Table 4 shows the comparison of detection accuracy among the three detectors for images with large resizing ratios. Obviously, the accuracy of the proposed approach is obviously higher than that of other methods.

### 5.3. The Detection of Images with Mixed Resizing Ratios

In order to verify the universality and robustness of this approach, the images with small or large resizing ratios are mixed together for the experiment. There is no significant difference between the true positive rate and the true negative rate for the same resizing ratio, and the results show that the detector's ability to detect tampered images is balanced with the ability to detect normal images, as shown in Table 5.

From Table 6, the detection accuracy of the proposed approach is superior to the other approach when the resizing ratio is over or equal to 5%, and it is much higher than the other two approaches when the resizing ratio is over or equal to 7%. It shows that the proposed approach is also robust on mixed resizing ratios.

**Figure 7:**
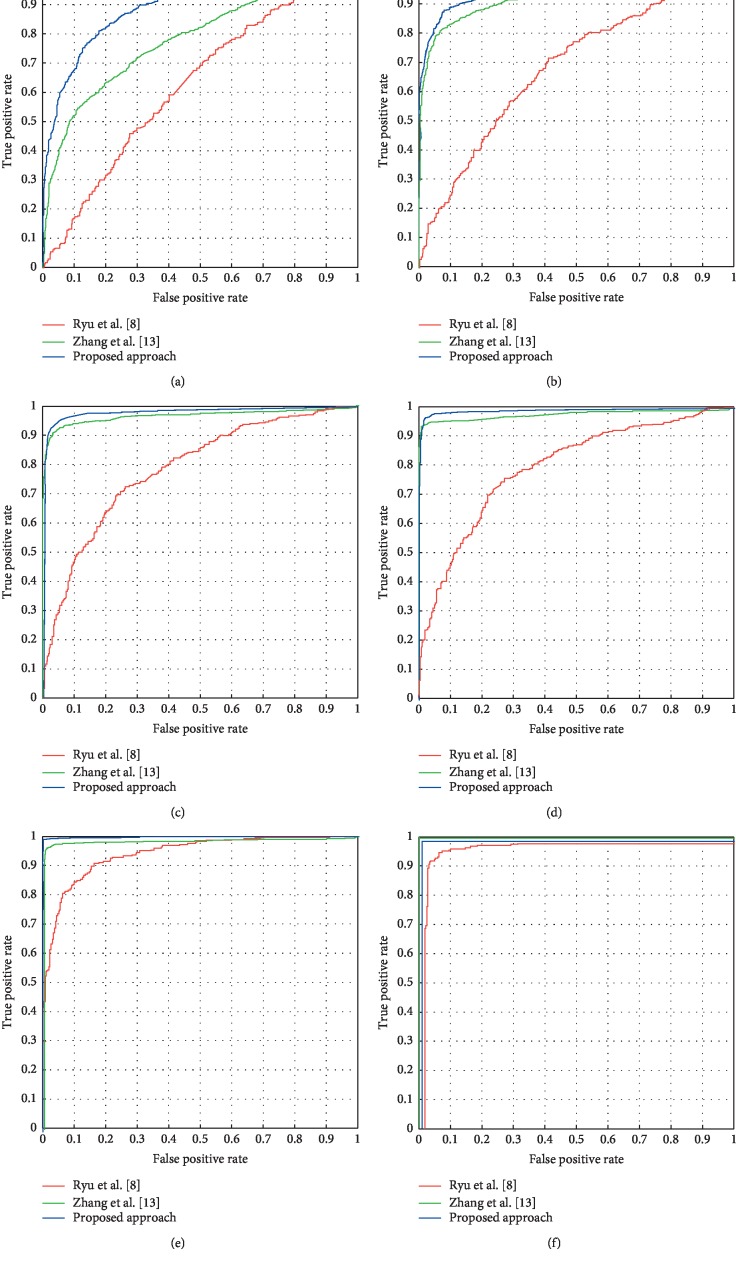
The contrast of ROC curves among the three detectors. Resizing ratio of (a) 5%; (b) 9%; (c) 11%; (d) 20%; (e) 30%; (f) 50%.

Figures 7(a)to 7(f) show the ROC curves [21] when the resizing rate is 5%, 9%, 11%, 20%, 30%, and 50%, respectively. The AUC (area under curve) [21] of the ROC curve of the proposed approach is higher than that of the other two approaches, and it shows that the proposed approach has the strongest detection ability. The proposed approach obtains the best performance among the three detectors, when the resizing ratio exceeds 5%. Moreover, the stability detection accuracy for the images seam carved can be achieved, when the resizing ratio is more than 11%.

## 6. Conclusion

Seam carving is a widely used content-aware image resizing technology. However, it can also be used to forge images. How to detect images seam carving becomes an important part of digital image forensics. In this paper, we propose a novel binary pattern called local neighborhood magnitude occurrence pattern (LNMOP) for the detection of seam carving. In order to compensate for the lack of magnitude information by conventional local descriptors, LNMOP extracts local features from the magnitude distribution of local neighborhood pixel intensity difference. On this basis, we propose an approach for the detection of seam carving based on LNMOP and HOG. Firstly, the HOG feature level is determined, and then the final features are selected according to the HOG feature level. Finally, the classifier is trained and tested by the final features. The performance of detection is also better for relatively smooth region, due to that different detection radius are used. In order to select better final features from the extracted LNMOP features, a feature selection algorithm based on HOG feature hierarchical matching is proposed, which reduces the dimension of LNMOP and reduces the computation of classifier. The experimental results show that the proposed approach can achieve better detection performance compared with other approaches. However, the proposed approach does not consider the postprocessing of tampered images. For example, a seam-carved image is JPEG-compressed or inpainted. For future work, we will explore a better approach to solve the forensics problem for the postprocessed seam-carved image.

## Figures and Tables

**Algorithm 1:**
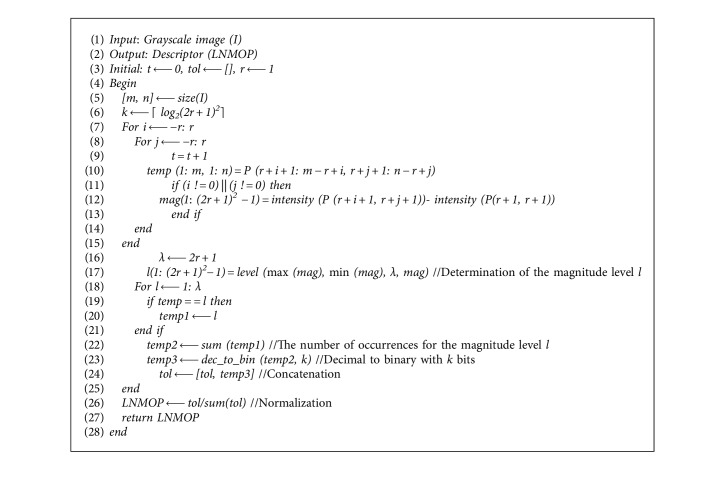
The LNMOP descriptor construction.

**Algorithm 2:**
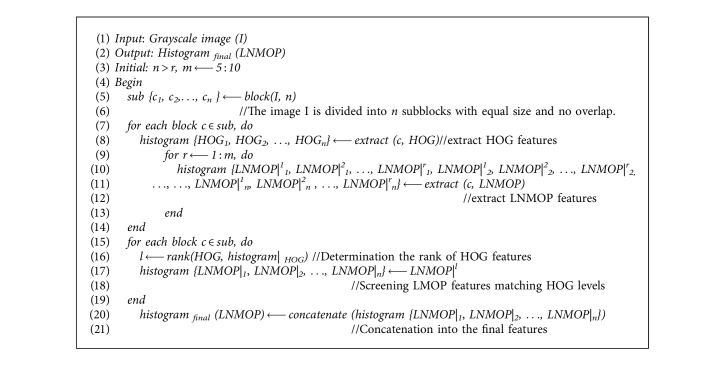
The algorithm of feature selection.

**Table 1:** The detection performance for images with small resizing ratios.

Resizing ratio (%)	True positive rate (%)	True negative rate (%)	Accuracy (%)
3	69.32	73.76	71.54
5	78.33	81.25	79.79
7	83.11	86.39	84.75
9	85.85	90.69	88.27
11	93.25	92.79	93.86

**Table 2:** The comparison of detection accuracy among the three detectors for images with small resizing ratios.

Resizing ratio (%)	Ryu et al. [8] (%)	Zhang et al. [13] (%)	Proposed approach (%)
3	54.56	64.28	71.54
5	57.83	75.12	79.79
7	59.01	78.32	84.75
9	61.22	80.87	88.27
11	93.61	94.11	93.86

**Table 3:** The detection performance for images with large resizing ratios.

Resizing ratio (%)	True positive rate (%)	True negative rate (%)	Accuracy (%)
10	90.25	89.99	90.12
20	95.78	93.94	94.86
30	96.98	94.90	95.94
40	99.60	97.30	98.45
50	99.96	98.30	99.13

**Table 4:** The comparison of detection accuracy among three detectors for images with large resizing ratios.

Resizing ratio (%)	Ryu et al. [8] (%)	Zhang et al. [13] (%)	Proposed method (%)
10	65.41	83.53	90.12
20	75.78	93.92	94.86
30	85.88	95.94	95.94
40	91.96	97.57	98.45
50	96.39	98.96	99.13

**Table 5:** The detection performance for mixed resizing ratios.

Resizing ratio (%)	True positive rate (%)	True negative rate (%)	Accuracy (%)
3	35.03	35.21	35.12
5	44.65	43.93	44.29
7	79.51	80.45	79.98
9	89.32	88.40	88.86
10	90.65	88.65	89.65
11	89.55	91.79	90.67
20	92.69	93.75	93.22
30	93.69	94.87	94.28
40	95.25	94.83	95.04
50	98.79	98.99	98.89

**Table 6:** The comparison of detection accuracy among three detectors for mixed sets.

Resizing ratio (%)	Ryu et al. [8] (%)	Zhang et al. [13] (%)	Proposed method (%)
3	35.28	31.54	35.12
5	38.92	43.79	44.29
7	42.32	49.75	79.98
9	46.13	60.02	88.86
10	48.78	65.32	89.65
11	49.87	70.27	90.67
20	62.13	88.02	93.22
30	84.02	93.45	94.28
40	84.34	94.84	95.04
50	98.78	98.80	98.89

## Data Availability

The data used to support the findings of this study are included within the article. Previously reported data were used to support this study and are available in the relevant references. These prior studies (and datasets) are cited at relevant places within the text as references [1–15, 17–21]. The processed data are available from the corresponding author upon request.
